# The topical application of low-temperature argon plasma enhances the anti-inflammatory effect of Jaun-ointment on DNCB-induced NC/Nga mice

**DOI:** 10.1186/s12906-017-1850-9

**Published:** 2017-06-27

**Authors:** Jeong-Hae Choi, Yeon-Suk Song, Hae-June Lee, Gyoo-Cheon Kim, Jin-Woo Hong

**Affiliations:** 1 Department of Internal Medicine, School of Korean Medicine, Pusan National University, Yangsan, 626-870 South Korea; 2 Department of Anatomy and Cell Biology, School of Dentistry, Pusan National University, Yangsan, 626-870 South Korea; 3 Department of Electrical Engineering, Pusan National University, Busan, South Korea; 4 (Bio)medical Research Institute, Pusan National University Hospital, Busan, South Korea

**Keywords:** Low-temperature argon plasma, Transdermal drug delivery of herbal extracts, Jaun-ointment, *Angelica gigas* Nakai, *Lithospermum erythrorhizon* Siebold & Zucc, DNCB-induced cutaneous inflammation in NC/Nga

## Abstract

**Background:**

Jaun-ointment (JO), also known as Shiunko in Japan, is one of the most popular medicinal formulae used in Korean traditional medicine for the external treatment of skin wound and inflammatory skin conditions. Since JO is composed of crude mixture of two herbal extracts (radix of *Lithospermum erythrorhizon* Siebold & Zucc and *Angelica gigas* Nakai), those been proved its anti-inflammatory activities *in-vitro* and *in-vivo*, JO has been expected as a good alternative treatment option for atopic dermatitis (AD). However, due to the lack of strategies for the penetrating methods of JO’s various anti-inflammatory elements into the skin, an effective and safe transdermal drug delivery system needs to be determined. Here, low-temperature argon plasma (LTAP) was adopted as an ancillary partner of topically applied JO in a mice model of AD and the effectiveness was examined.

**Methods:**

Dorsal skins of NC/Nga mice were challenged with DNCB (2,4-dinitrochlorobenzene) to induce AD. AD-like skin lesions were treated with JO alone, or in combination with LTAP. Inflammatory activity in the skin tissues was evaluated by histological analysis and several molecular biological tests.

**Results:**

LTAP enhanced the effect of JO on AD-like skin lesion. Topical application of JO partially inhibited the development of DNCB-induced AD, shown by the moderate reduction of eosinophil homing and pro-inflammatory cytokine level. Combined treatment of JO and LTAP dramatically inhibited AD phenotypes. Interestingly, treatment with JO alone did not affect the activity of nuclear factor (NF)κB/RelA in the skin, but combined treatment of LTAP-JO blocked DCNB-mediated NFκB/RelA activation.

**Conclusions:**

LTAP markedly enhanced the anti-inflammatory activity of JO on AD-like skin lesions. The effect of LTAP may be attributed to enhancement of drug penetration and regulation of NFκB activity. Therefore, the combination treatment of JO and LTAP could be a potential strategy for the treatment of AD.

## Background

Atopic dermatitis (AD) is one of the major inflammatory skin diseases, characterized by hypersensitivity against various types of antigens [1]. Despite the increasing prevalence of AD [2], there is no known cure for AD. However, treatments can reduce the severity and control the symptoms, and are divided into three groups: anti-inflammatory drugs, skin-barrier reconstructing creams, and physical approaches. Currently, anti-inflammatory drugs such as corticosteroids and calmodulin inhibitors are considered as the first line of treatment, owing to their established effectiveness [3]. These drugs control the development of AD by inhibiting several immune responses. However, the risk of prolonged use of these drugs has been demonstrated in several studies [4, 5] and they are used with caution nowadays. Skin-barrier reconstructing creams minimize the exposure of the sensitive skin to allergens by enhancing the disrupted skin barrier of AD patients [6]. Therefore, skin-barrier reconstructing cream itself cannot reduce the inflammatory reactions associated with AD. One of the recent and upcoming strategies for treating AD is the use of medical UV devices [7–9]. These devices can reduce the exaggerated immune response of AD skin lesion. However, the use of UV in AD patients is accompanied by the risk of genetic mutations [10]. Therefore, combination treatments of anti-inflammatory drugs with other therapeutic options are frequently adopted to minimize drug dosage and risk associated with chronic use [11, 12].

Jaun-ointment (JO), which is also known as Shiunko, is one of the most frequently used traditional-alternative external medicines for treating various types of skin conditions, such as abrasions, cuts, frostbite and burns in Korea and Japan [13, 14]. In early clinical study of JO, JO improved the AD patients with anti-bacterial effects of JO, but anti-inflammatory effects was neglected [15]. Recently, JO was adopted to control other skin inflammatory conditions [16], but still need to be improved its clinical efficacy. The major component of JO is a mixture of crude extracts of *Angelica gigas* radix and *Lithospermum erythrorhizon* radix, which are widely known for their anti-inflammatory properties [17, 18] and wound healing ability [19, 20]. Many functional components of the extracts have been investigated as candidate drugs in several inflammatory diseases, including AD. For example, shikonins and lithospermic acid isolated from the extract of *L. erythrorhizon* radix have been examined for their anti-inflammatory activity [21–23]. Furthermore, decursin from the extract of *A. gigas* radix has been reported to be a potential modulator of immune reactions [24, 25]. For these reasons, the extracts of *A. gigas* radix and *L. erythrorhizon* radix have been used to target AD, and it has been reported that several immune responses were affected by the oral administration of these extracts in mice [26, 27].

Low-temperature atmospheric-pressure plasma (LTAPP), or non-thermal plasma and tissue-tolerable plasma in other terms, is a highly active ionized gas, which has attracted significant interests in the field of dermatology [28]. Since LTAPP simultaneously ejects several functional elements during its generation step, it is believed to regulate several biological reactions. LTAPP not only stimulates wound healing and skin surface decontamination [29, 30], but has also been suggested to be used for the cancer treatments [31, 32]. Furthermore, our previous study and the findings of Lademann et al., highlighted the safe and efficient transdermal drug delivery using LTAPP [33, 34]. Unlike other physical drug delivery systems, LTAPP can enhance drug absorption by temporary scattering cell-to-cell bonding in the epidermis, without harming tissues. This property of LTAPP might enhance the therapeutic effects of many topically applied drugs.

In this study, low-temperature argon plasma (LTAP), a type of LTAPP, was employed to test whether the combinational treatment of LTAP with JO can enhance the anti-inflammatory effect of JO in AD mice model. Firstly, the effect of topical JO on AD was determined in a mouse model of AD induced by 2,4-dinitrochlorobenzene (DNCB) and the effect of JO was compared to that of combination treatment of LTAP and JO (LTAP-JO). The clinical efficacy was evaluated by the dermatitis scores, and several histological and molecular biological analyses of skin tissues. Furthermore, to explore the LTPA-JO’s mechanism of action, the activity of nuclear factor (NF) κB in the skin lesion were monitored.

## Methods

### Reagents

All chemicals were obtained from Sigma-Aldrich Korea unless otherwise indicated.

### Jaun-ointment

**Fig. 1:**
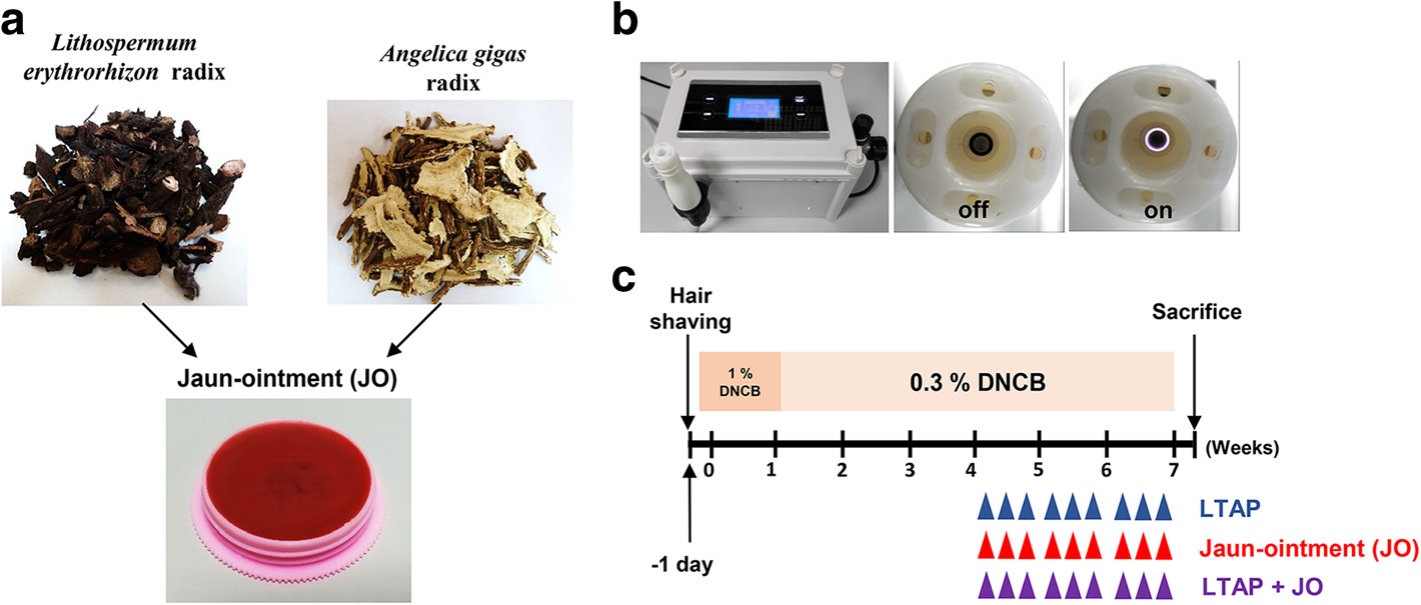
A brief introduction of Jaun-ointment (JO) and low-temperature argon plasma (LTAP) device and the experimental design. **a** Photographs showing the appearance of *Angelica gigas* Nakai radix and *Lithospermum erythrorhizon* Siebold & Zucc radix*,* two main components of JO. **b** The figures of LTAP device used. **c** Schematic diagram of the experimental protocol in a mouse model. NC/Nga mice were divided into four groups: non-treated control (nt), DNCB-induced AD-like symptoms (DNCB), DNCB-induced AD-like symptoms treated with JO (DNCB-JO), and DNCB-induced AD-like symptoms treated with LTAP and JO (DNCB-LTAP-JO)

JO in this study was a kind gift from the Hanpoong Pharm & Foods Corp. (Jaungo^Ⓡ^, Hanpoong Pharm & Foods Corporation, Seoul, Republic of Korea), which was manufactured and regulated according to the guidelines of the Korea Food & Drug Administration. In brief, the 606.1 g of Oleum Sesami was boiled for 1 h, and then the 202.7 g of Beeswax were added and boiled till melted completely. This mixture was used as a vehicle of JO in this study. The 60.6 g of *Angelica gigas* radix and the 72.7 g of *Lithospermum erythrorhizon* radix were added to the vehicle mixture and heated at temperature 140 °C on heating stirrer till the color turns into the red-violet. Then, the herbs were filtered with cotton patch, and let the mixture solution become the form of ointment on the moderate shaker at room temperature (Fig. 1a).

### LTAP device

For this study, a new low-frequency LTAP generator was developed (Fig. 1b). As a type of low-frequency (15 kHz) plasma device, this device ejects a single plasma flow with the temperature of 32 °C (at the end of the generator tip). The Vapp of the plasma-treating mode is about 3 kV, and 2 slm (standard litters per minutes) of pure argon gas was used as a vehicle gas. Unlike the device in our previous reports [34], this new device produces the plasma glow only within plasma producing aisle, so that the plasma can’t contact to the skin directly. However, the device helped transdermal drug delivery activity effectively (Data not shown).

### Animal preparation and procedure for the treatments

Six-week-old, pathogen-free, male NC/Nga mice were obtained from SLC, Inc. (Shizuoka, Japan). Animals were acclimatized to the room conditions (temperature of 22 ± 2 °**C** and humidity of 55 ± 5%) for at least 2 weeks prior to experiments. Mice were allowed free access to Purina rodent chow (Seoul, Korea) and tap water. At the beginning of experiments, body weight of the mice were 25 g on average. The mice were divided into 5 groups (*n* = 5 per group): non-treated control (nt), DNCB treatment only (DNCB), DNCB treatment with topical application of JO (DNCB-JO), DNCB with skin surface treatment of LTAP (DNCB-LTAP) and DNCB with combination treatment of JO and LTAP (DNCB-LTAP-JO). To induce AD-like skin lesions, DNCB was applied on the dorsal skin. After removal of the dorsal hairs of the mice (an area approximately 8 cm^2^) using an electric shaver, 200 μl of 1% DNCB solution dissolved in a mixture of olive oil and acetone (3:1) was applied on the shaved back skin 3 times a week for sensitization. After that, the skin was challenged with 200 μl of 0.3% DNCB solution 3 times per week for 6 weeks. Before the application of JO, LTAP and LTAP-JO (on day 29), visible AD symptoms in mice of the DNCB, DNCB-JO, DNCB-LTAP and DNCB-LTAP-JO groups were monitored briefly, and DNCB-induced AD was confirmed in all mice of the 4 groups. In the DNCB-JO group, 100 mg of JO was topically applied on the dorsal skin of anesthetized mice. For DNCB-LTAP group, the dorsal skin of the mice were treated with LTAP for 5 min. In the DNCB-LTAP-JB group, the mice was exposed to LTAP for 5 min just before the topical application of JO. The distance from the target skin to the flair end of LTAP was 5 mm, determined by the spacer of the device. As shown in Fig.1c, these procedures were repeated for 3 weeks (3 times a week), after the treatment of DNCB. All mice were sacrificed on day 50 after the first application of DNCB. Four equal sized skin tissues from each mouse were acquired using a 6-mm biopsy punch (Militex) for the following experiments.

### Evaluation of skin dermatitis

The severity of dermatitis was evaluated once a week, just before the last DNCB application of the week. Development of dryness/scarring, edema, erythema/hemorrhage, and erosion/excoriation were scored according to the severity: 0 (none), 1 (mild), 2 (moderate), or 3 (severe). The sum of the 4 individual scores was defined as the dermatitis score [35].

### Skin thickness analysis

Under a light microscope, measurements of skin thickness were performed using the i-Solution Lite software (IMT i-Solution, Vancouver, BC, Canada). Ten readings were obtained from each epidermis of each animal. The epidermal thickness was measured from the stratum basal to the stratum granulosum (excluding the stratum corneum).

### Histology, immunohistochemistry, and immunofluorescence assay

Standard histological paraformaldehyde fixation, paraffin embedding, and immunostaining were performed. Briefly, the skin biopsy samples were fixed in 4% paraformaldehyde for 24 h, and then embedded in paraffin. Sections of the skin (5 μm) were stained with hematoxylin-eosin (H&E) or toluidine blue to monitor the histological changes in the skin and recruitment of mast cell, respectively. Eosinophil peroxidase (EPX) staining was performed using a goat polyclonal anti–EPX antibody (Santa Cruz) and a ABC alkaline phosphatase staining system (Vector Laboratories) with 3,3′diaminobenzidine (DAB) as the staining substrate. NFκB/RelA staining within the skin tissue was performed using a rabbit monoclonal anti–NFκB/RelA antibody (Cell signaling technology) and Alexa Fluor 594 goat anti-rabbit antibody (Invitrogen). The nuclei within the tissues were counterstained using hematoxylin or 4′,6-diamidino-2-phenylindole (DAPI).

### Reverse transcription polymerase chain reaction (RT-PCR) analysis.

The skin tissues of mice and isolated skin biopsy sample were sliced into tiny pieces. Then, TRIzol reagent was added and grinded with a handy tissue grinder (BioMasher® coupled with pestle motor) until no visible tissues remained in the reagents. RT-PCR analysis was performed according to the method of previous study [36]. The primer sequences used were specific primers against mouse thymus and activation-regulated chemokine (TARC, sense: AGGGCAAGCTCATCTGTGC, antisense: GGGAGGAAGGCTTTATTCCG), eotaxin (sense: CCAATTCGATCCCCTGTCA, antisense: CCCCTCAGCTCAGTGTGG) and GAPDH (sense: ACTGGCATGGCCTTCCGT, antisense: CCACCCTGTTGCTGTAGCC), respectively.

### Western blot analysis

Total protein was extracted from the skin tissues using a tissue grinder in the presence of protein lysis buffer. Ice-cold lysis buffer (1.5 mL, 50 mM Tris/HCl, pH 7.5, 150 mM NaCl, 1% (*v*/v) Nonidet P40, 10% (*v*/v) glycerol, 1 mM PMSF, 1 mM dithiothreitol, 20 mM NaF, and 1 mM EDTA containing 1 × protease inhibitor cocktail (Roche)) per skin biopsy was used. Protein lysate (30 μg) was resolved by sodium dodecyl sulfate polyacrylamide gel electrophoresis (SDS/PAGE) gel (8–10%) and transferred to polyvinylidene difluoride (PVDF) membranes (Millipore). Upon the completion of transfer, the membranes were probed with the antibodies against p65/RelA and phospho-p65/RelA (Cell Signaling technology), p50 and phosphor-p50 (Santa-Cruz biotechnology). The bands were visualized with Advanced ECL® Western Blotting Detection Reagents (Amersham Biosciences).

### Enzyme-linked immunosorbent assay (ELISA)

Protein levels of Immunoglobulin E (IgE), chemokine (C-C motif) ligand 17 (CCL17), and interferon gamma (IFNγ) were determined from the protein extracts of the 6-mm punch biopsy skin specimens. After measuring the protein concentration of each sample, equal amount of total protein solutions were subjected to an ELISA according to the manufacturer’s instructions (IgE and IFNγ: KOMA biotech, CCL17: R&D systems). The levels of the cytokines and IgE were normalized to the weight of the tissue specimen, and the data were presented as the relative protein level.

### Data analysis

Data were presented as means ± standard error of the mean (SEM) of at least 4 independent experiments. Two-tailed Student’s *t*-tests were used to assess statistical significance for differences in means, and the significance was set at *P* < 0.05. The significance and validity of the data were confirmed further by performing one-way analysis of variance (ANOVA).

## Results

### The effects of LTAP-JO treatment on DNCB-induced AD-like symptoms

**Fig. 2:**
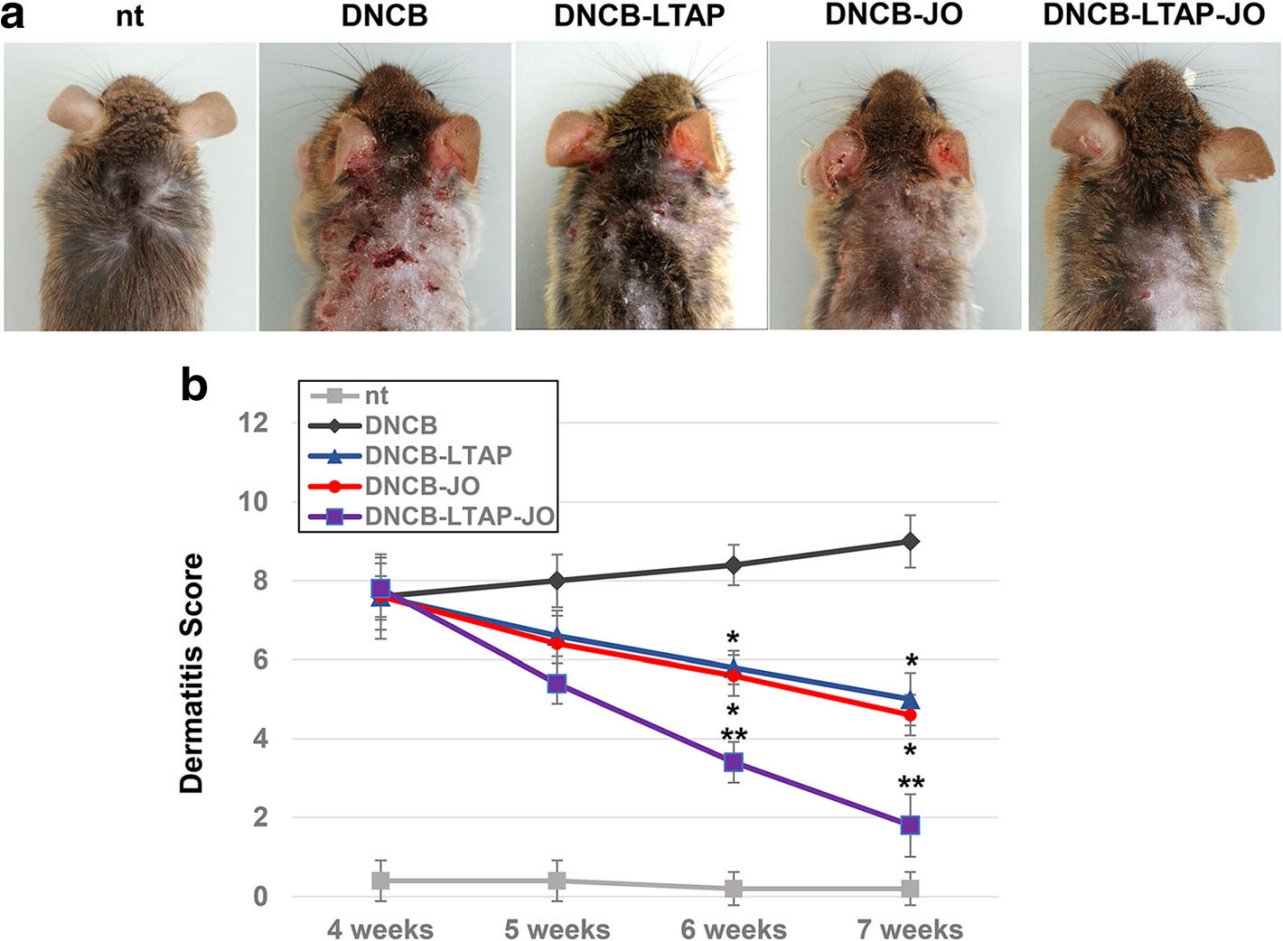
The effects of LTAP-JO treatment on the clinical severity of DNCB-induced AD-like symptoms in mice. **a** Images of the dorsal lesions from the mice just after the sacrifice. **b** The dermatitis scores were evaluated weekly between the end of week 4 and week 7. Data represents the mean ± SEM (*n* = 5). Means with the symbols (*, **) indicate significantly different from each other (against DNCB and DNCB-JO or DNCB-LTAP respectively) at *p* < 0.05

To evaluate the beneficial effect of JO and LTAP-JO treatment on DNCB-induced AD-like symptoms in mice, the dermatitis scores were determined at different durations of the experiment, and morphological changes of the skin were monitored. As shown in Fig. 2a, repeated application of DNCB induced tiny wounds with the trace of hemorrhage on the dorsal, but the topical administration of JO alone slightly reduced of the number of wounds and the intensiveness of hemorrhage and scars. Interestingly, the use of LTAP alone also showed moderate decrease of DNCB-mediated symptoms. As we expected, LTAP-JO treatment completely inhibited DNCB-mediated external changes of the skin. Fig. 2b shows the average dermatitis score, which represents the severity of the overall clinical symptoms of AD, including erythema, hemorrhage, edema, scarring, and erosion. Four weeks of DNCB treatment increased the dermatitis score to 7.6 on an average, and the score was 9 at the end of the experiments. The dermatitis score was moderately decreased with the treatment of JO (scored 4.6 in the end). The effectiveness of LTAP treatment was quite similar to that of JO treatment (scored 5 in the end). Finally, the score of the mice treated with LTAP-JO rapidly decreased and a score of 1.8 was determined after 3 weeks of treatment.

### The effect of LTAP-JO treatment on DNCB-induced histological changes in the dorsal skin

**Fig. 3:**
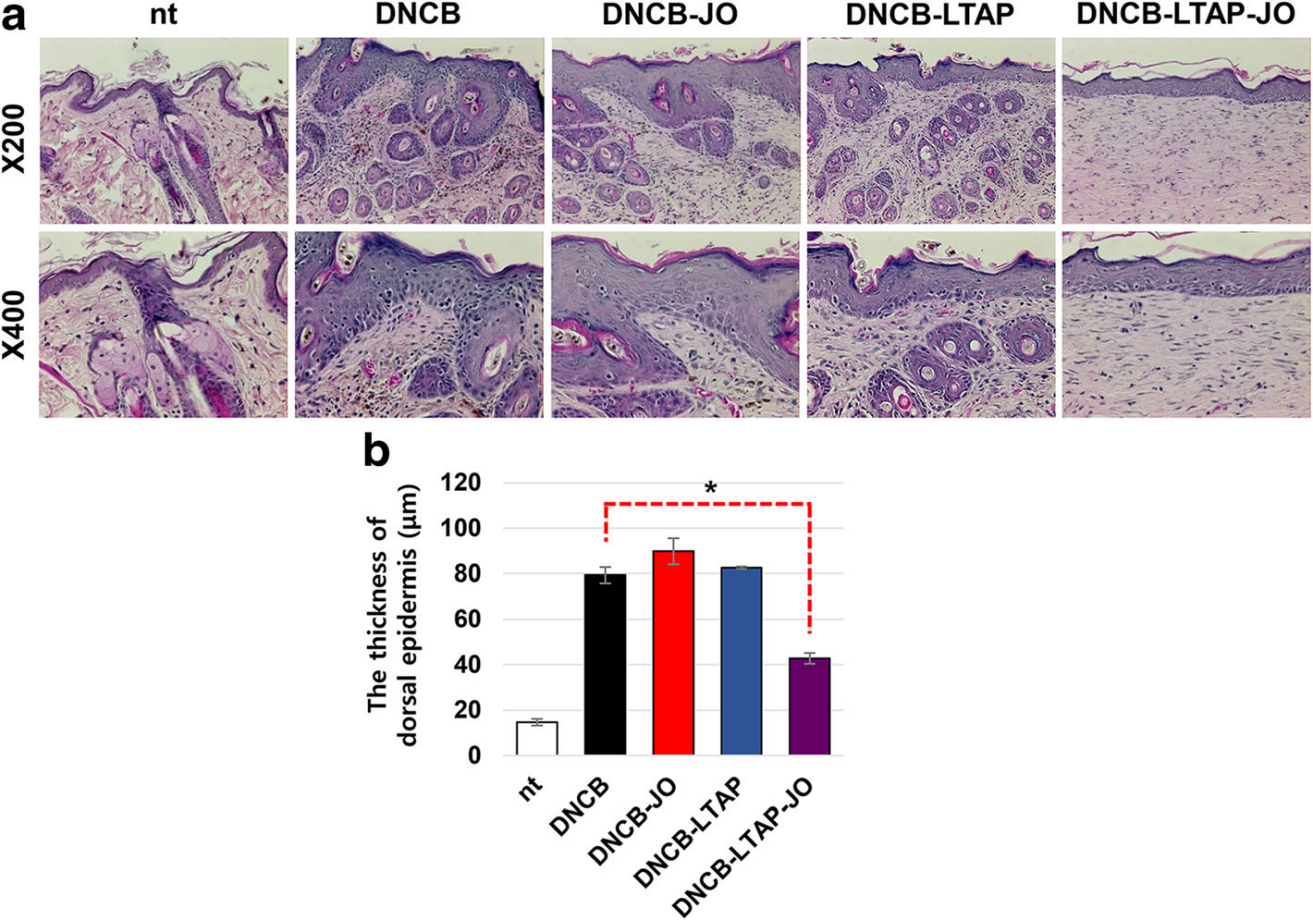
The effects of LTAP-JO treatment on DNCB-induced changes in the dorsal skin tissue. **a** The results of H&E staining using dorsal skin tissues isolated from the mice of each group at the end of the experiments. Data shown are the representatives of each group of mice. **b** The epidermal thickness of the dorsal skin was measured using an image analysis system as described in 2. Materials and Methods. Data represents the mean ± SEM (*n* = 50). The significance of the difference (*p* < 0.05) is described as*

To further understand the role of LTAP-JO on DNCB-induced AD-like symptoms in mice, the skin tissues of mice were employed for histological analysis. First, H&E staining was performed to elucidate the effect of LTAP-JO treatment on the overall skin structures. As shown in Fig. 3a, repeated treatment of DNCB increased the dermal cell population in the dorsal skin; in particular, cells with round-shaped nucleus (stained in purple) were greatly accumulated. JO treatment slightly decreased the dermal cell population in the skin lesion. The mere treatment of LTAP also reduced dermal cell population in moderately manner, but LTAP-JO treatment decreased the dermal cell population further. Although the overall dermal cell population was not greatly reduced by LTAP-JO, DNCB-mediated induction of round-shaped nuclei cells was significantly inhibited by LTAP-JO. A thick epidermal layer is one of the major characteristics of AD skin lesion. As shown in Fig. 3b, DNCB increased the epidermal thickness approximately 5.2-fold of the non-treated skin. The treatment with JO or LTAP alone did not affect DNCB-mediated skin thickening along with the mere treatment, but combination treatment of JO and LTAP reduced the epidermal thickness to half of that of the DNCB-treated skin. Skin damage such as physical destruction of skin tissues was not detected after LTAP-JO treatment.

### The effect of LTAP-JO treatment on DNCB-mediated accumulation of mast cell and eosinophil in the skin

**Fig. 4:**
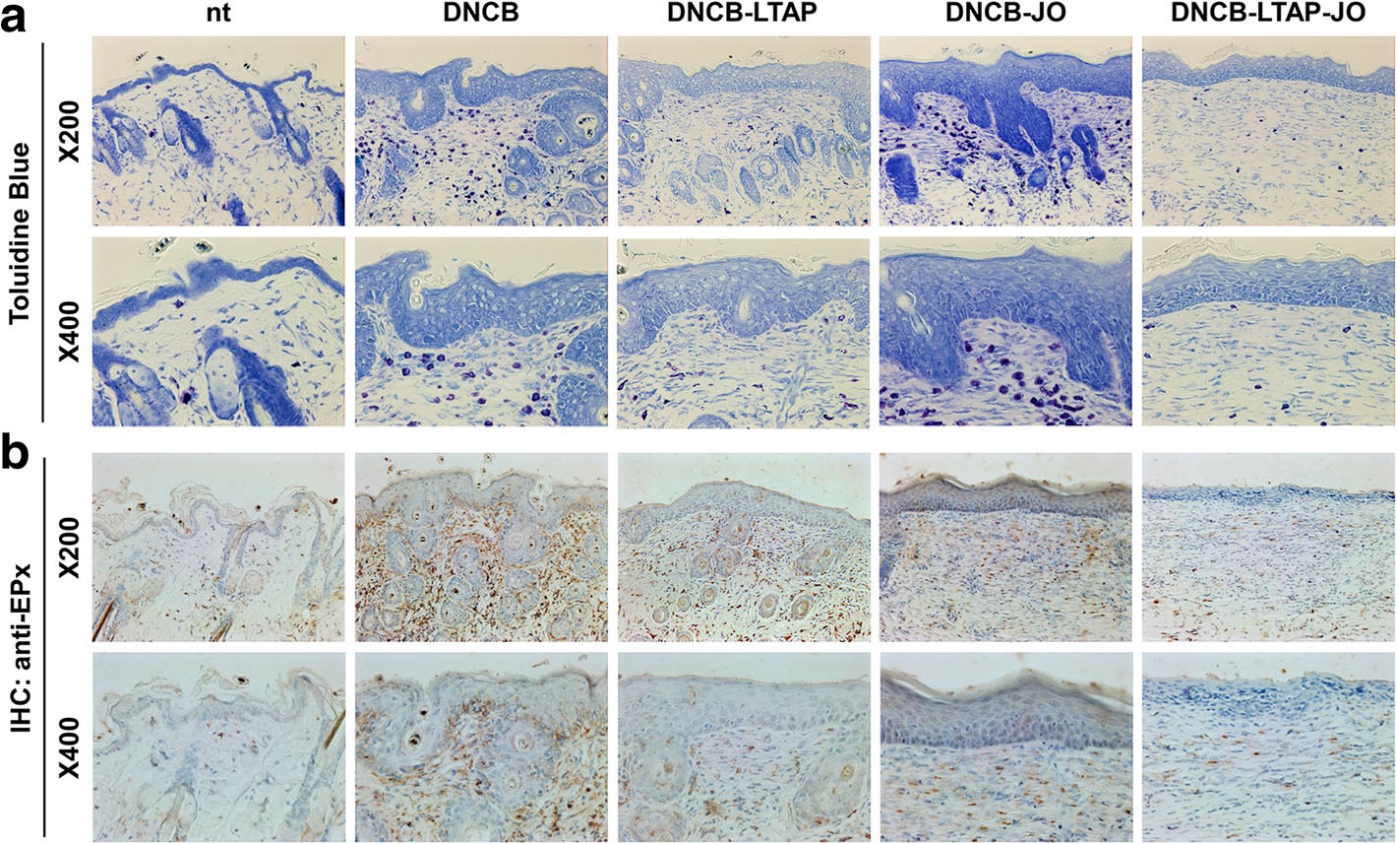
The effects of LTAP-JO treatment on DNCB-mediated accumulation of mast cell and eosinophil in the skin. **a** The result of toluidine blue staining of the skin tissues of mice from each group. The mast cells are shown as dark-purple dots in the dermis. **b** The result of the immunohistochemistry assay against eosinophilic peroxidase (EPX). Eosinophils are shown as stained cells in brown. The nuclei of the cells in skin tissues are counterstained with hematoxylin. Data shown are representatives of each group of mice (*n* = 5)

To determine the effect of JO and LTAP-JO treatment on the inflammatory reaction of AD-like skin lesion, the infiltrated immune cells within the lesions were monitored. Toluidine blue staining method is one of the popular tools for visualizing the mast cells within skin sections. As shown in Fig. 4a, DNCB treatment increased the number of mast cells in the skin lesion, especially in the dermal area near the basal lamina. Interestingly, the treatment of DNCB-induced AD-like lesion with JO did not affect the number of mast cells. On the other hand, LTAP treatment partially blocked DNCB-mediated mast cell accumulation, and LTAP-JO treatment significantly reduced the number of mast cells in the lesion. Meanwhile, eosinophil infiltration is a histological marker of AD. In contrast to the effect of JO on mast cell, JO treatment alone markedly reduced DNCB-mediated increase of eosinophil in the dermis. The effect of LTAP on eosinophil accumulation was similar to JO treatment, and LTAP-JO treatment further decreased the dermal eosinophil population (Fig. 4b).

### The effect of LTAP-JO on DNCB-mediated molecular biological changes in the skin lesion

**Fig. 5:**
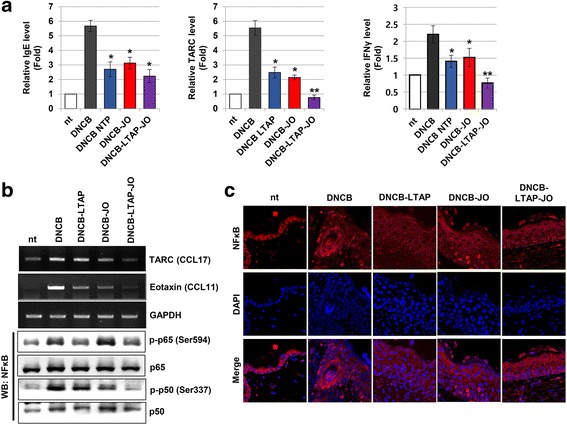
The effect of LTAP-JO on DNCB-mediated molecular and biological changes in the skin lesion. (**a**) The levels of IgE, TARC, and IFNγ in the dorsal skin tissues of mice were measured using an ELISA kit. Data are shown as the relative level of the proteins, and represents the mean ± SEM (*n* = 5). Means with the symbols (*, **) indicate significant differences (*p* < 0.05). (**b**) The result of the RT-PCR and Western Blot assay using the skin tissues. Data shown are representatives of 4 independent experiments. (**c**) The location of NFκB in the skin tissue was monitored by an immunofluorescence assay against NFκB. Photographs were taken under a confocal microscope at 400× magnification. Data shown are the representatives of each group of mice

Several pathological marker proteins could indicate the severity of inflammation in the skin lesion. To determine the therapeutic activity of JO and LTAP-JO on AD, ELISA against IgE, TARC, and IFNγ was adopted (Fig. 5a). The repeated treatment with DNCB increased the protein levels of IgE and TARC, approximately 5.7 and 5.5 fold respectively, but only a 2.2-fold increase in IFNγ was observed. Treatment of DNCB-induced AD skin lesion with JO decreased the levels of IgE, TARC, and IFNγ by 3.1-, 2.1-, and 1.5-fold, respectively. The effect of LTAP treatment was very similar to that of JO. More importantly, LTAP-JO treatment completely inhibited DNCB-mediated induction of TARC and IFNγ, but a small reduction in IgE level (2.1-fold) was observed.

Various types of non-immune cells including keratinocyte in the epidermis and fibroblast in the dermis play important roles in the development of AD by producing and secreting various pro-inflammatory cytokines. To confirm whether the blockage of immune cell homing after treatment with JO and LTAP-JO was related to the production of cytokines involved in immune cell homing in the skin lesion, RT-PCR was performed using the skin tissues. As shown in Fig. 5b, the independent treatment of JO and LTAP showed moderate of DNCB-mediated upregulation of mRNA expression of eotaxin and TARC, but LTAP-JO treatment inhibited DNCB-mediated induction completely. Since the importance of NFκB in several immune responses of AD has been reported, the effect of JO and LTAP-JO on NFκB activity in the lesion was monitored. Interestingly, the total protein level of p65 was not affected by all three treatment methods, but DNCB-induced phosphorylation at Ser 536 residue decreased in mice treated with LTAP and LTAP-JO. The treatment of JO alone shoed slight increase of p65 phosphorylation. On the other hand, the treatment with JO reduced the phosphorylation of p50 (Ser 337) moderately, but the LTAP treatment showed the less effectiveness than JO. The LTAP-JO treatment completely blocked the DNCB-mediated phosphorylation of p50. The activity of NFκB in the lesion was examined further using an immunofluorescence assay. As shown in Fig. 5c, DNCB stimulated nuclear localization of NFκB in the cells of the epidermis. The treatment of JO itself showed limited effects on the nuclear localization of NFκB in the upper layers of the epidermis, but LTAP alone and LTAP-JO treatment completely inhibited DNCB-mediated nuclear localization of NFκB throughout the epidermis and dermis.

## Discussion

The skin barrier limits the clinical efficacy of herbal extracts despite several functional components in the extract [37]. The skin blocks spontaneous internalization of compounds into the body because of their hydrophobicity and size. Medicinal herbal extracts contain a broad spectrum of active components that have difficulty penetrating through the skin barriers. Most external medicines used in traditional Korean medicine, including JO, are associated with penetration issues, owing to a limited number of strategies established for enhancing the absorption of the functional components. In our previous study, the enhancing property of LTAP for transdermal drug delivery was elucidated. The treatment of LTAP effectively enhanced the absorption of epidermal growth factor by temporarily disturbing the cellular junction-mediated skin barrier function. Here, this property of LTAP was adopted to further evaluate the anti-inflammatory effect of JO in a mouse model of AD.

DNCB induced inflammatory reactions on the dorsal skin, and the clinical effects of JO and LTAP-JO were first determined by monitoring the clinical symptoms of dermatitis (Fig. 2). Although JO or LTAP treatment alone slightly decreased DNCB-induced dermatitis (as shown by the dermatitis score), pre-treatment of LTAP further enhanced the therapeutic effect of JO and strongly inhibited DNCB-mediated AD phenotypes. The effect of combination treatment with LTAP and JO was further confirmed by histological analysis of the skin tissues. Increased epidermal thickness and increase in the number of dermal cells are the major characteristics of AD skin lesion [38], which were induced by repeated treatment of DNCB (Fig. 3). Interestingly, JO or LTAP alone failed to reduce DNCB-induced thickening of the epidermis, but LTAP-JO treatment inhibited (approximately 50%) the effect of DNCB. Furthermore, DNCB-mediated increase of dermal cells was slightly impeded by the topical application of JO, and LTAP-JO treatment strongly inhibited the increase of dermal cells. Further, the treatment of JO specifically blocked the increase of eosinophil, but did not affect the population of mast cells. On the other hand, LTAP treatment alone showed much efficient on blocking mast cell accumulation with similar effectiveness on eosinophil. LTAP-JO treatment inhibited DNCB-mediated accumulation of both cells significantly. These data suggest that some anti-inflammatory components within JO can spontaneously penetrate into the skin and partly controls DNCB-mediated immune responses, such as increase of eosinophils. Hence, LTAP might enhance the penetration of several components in JO, and in addition, since LTAP itself also has some anti-inflammatory activity, LTAP-JO treatment showed significant inhibition of DNCB-mediated inflammation.

The effects of LTAP-JO on AD were further explored and compared to the effects of JO or LTAP alone. The levels of several AD marker proteins in the lesion were monitored (Fig. 5a). JO or LTAP treatment alone feebly reduced DNCB-mediated elevation of IgE, TARC, and IFNγ. LTAP-JO treatment completely inhibited the induction of TARC and IFNγ but did not affect IgE. TARC is a type-2 T helper cell (Th2 cell) attracting cytokine, which is very important in the early stage of immune reactions of AD. TARC stimulates recruitment of Th2 cells in the lesion [39], leading to Th2 cell-driven immune reactions, including the recruitment of mast cells and eosinophils, and the maturation of IgE producing B-cells. The level of IgE is one of the most important markers of AD because IgE can stimulate important immune reactions in the lesion by triggering bulk cytokine release from immune cells, including mast cells. The increase of IFNγ is a major feature of the late stages of Th1 cell-driven AD immune reactions, which leads to an exaggerated immune response and epidermal thickening in the AD lesion [40]. Therefore, the fact that LTAP-JO treatment effectively blocked DNCB-mediated induction of these 3 proteins indicates that the early and late immune responses of AD were effectively inhibited. The results of the RT-PCR analysis against TARC supported the result of the ELISA (Fig. 5b). More interestingly, DNCB-mediated production of eotaxin, which plays a pivotal role in the accumulation of eosinophil in the lesion [41], was slightly inhibited by JO or LTAP, but DNCB-mediated induction of eotaxin was completely inhibited by LTAP-JO treatment. This result was in accordance with the findings of the tissue-staining assay against eosinophils. Furthermore, a study has determined the effect of shikonin on the recruitment of eosinophil and eotaxin in an ovalbumin induced asthma-like model [22]. The immunological reactions of AD and asthma are similar and shikonin is the major component of JO; these findings are parallel with each other even though the immunological trigger and the location of the immune reactions are different.

Many kinds of transcription factors have been reported to play key roles in the development of AD, and NFκB is one of the major targets of AD treatment. NFκB plays a pivotal role in several types of immunological reactions, including lymphocyte maturation [42] and cytokine/pathogen-induced response of non-immune cells [43, 44]. Several anti-inflammatory drugs including glucocorticoids and FK-506, have been reported as regulators of NFκB activity [45]. Furthermore, studies have demonstrated that the anti-inflammatory components of *A. gigas* and *L. erythrorhizon* inhibited the activity of NFκB in various types of cells [21, 24]. As shown in Fig. 5b, the topical application of JO on AD-like lesion rarely affected DNCB-mediated phosphorylation of p65 subunit at Ser536, which is located at trans-activation domain of p65 and well known for the NFκB activity [46, 47]. On the other hand, LTAP and LTAP-JO treatment significantly inhibited DNCB-mediated p65 phosphorylation. Interestingly, JO treatment significantly reduced the phosphorylation of other subunit of NFκB, p50, which is very important for DNA binding activity of NFκB. LTAP treatment alone did not affected on the phosphorylation of p50, but LTAP-JO treatment showed complete blockage of DNCB-mediated p50 phosphorylation. This data represents that the molecular mechanisms of LTAP and JO mediated NFκB inhibition are different to each other. This effect was also observed when monitoring the localization of NFκB in skin tissues. As a transcription factor, NFκB has to enter the nucleus after activation. Treatment of JO alone did not affect DNCB-mediated NFκB translocalization, but LTAP-JO treatment showed successful withdrawal of NFκB from the nucleus (Fig. 5c). Several studies have established the importance of NFκB on B-cell mediated IgE production [48], mast cell activation [49], and production of TARC and eotaxin [50, 51]. Our results suggest that the anti-inflammatory effects of LTAP-JO might be related to the inhibition of NFκB (Fig. 5b and c).

In general, hydrophilic molecules and molecules larger than 500 Da rarely penetrate the skin by itself [52]. Shikonin is one of the main anti-inflammatory components of JO and its low molecular weight (288.3 Da) indicates that it is suitable as an external drug for the treatment of skin diseases; however, its hydrophilicity can be a problem. Decursin (328.36 Da) from *A. gigas* is a hydrophobic and appropriately sized molecule suitable for skin penetration and has demonstrated anti-inflammatory activity [25]. Lithospermic acid (718.61 Da) is another main anti-inflammatory component of JO, which rarely pass through the skin barriers due to its size and hydrophilicity. In fact, most medicinal herbal extracts have many functional elements that are over 500 Da and hydrophilic. In this study, the treatment of JO alone showed limited clinical efficacy in a mice model of AD. Although JO contains several anti-inflammatory compounds, the topical application of JO alone on skin lesion is not sufficient to obtain significant clinical efficacy. Limited penetration of anti-inflammatory components of JO due to the skin barrier might be attributed to this results. Since the pre-treatment of LTAP on the skin can enhance transdermal drug delivering by temporary weakening the skin barrier, combination treatment of JO and LTAP might enhance the skin-penetrating ability of a broader range of anti-inflammatory elements of JO. Therefore, along with anti-inflammatory activity of LTAP itself, the enhancement of transdermal drug delivery after LTAP treatment may be a reason for the improved anti-inflammatory activity of JO on AD-like skin lesion.

## Conclusion

The topical application of JO partially affected the immunological responses of AD, but the application of LTAP enhanced the clinical activity of JO in a mouse model of AD. The combination treatment of JO and LTAP strongly inhibited pathogen-mediated NFκB activation, leading to a reduction in AD symptoms. Along with anti-inflammatory activity of LTAP itself, the enhanced delivery of anti-inflammatory components in JO after LTAP treatment possibly accounts for the improved AD symptoms. Since repeated treatment of LTAP did not cause tissue damage, this method can be regarded as safe. Further evaluation of the clinical safety of LTAP is required; nevertheless, we believe that LTAP could be effective in combination with other topical herbal drugs in the near future.

## Abbreviations

AD: Atopic Dermatitis
CCL: Chemokine (C-C motif) ligand
DAB: Diaminobenzidine
DAPI: 4',6-diamidino-2-phenylindole
DNCB: 2,4-dinitrochlorobenzene
ELISA: Enzyme-linked immunosorbent assay
EPX: Eosinophil peroxidase
H&E: Hematoxylin and Eosin
IFN: Interferon
IgE: Immunoglobulin E
JO: Jaun-ointment
LTAP: Low-temperature argon plasma
LTAPP: Low-temperature atmospheric-pressure plasma
NFκB: Nuclear Factor kappa B
TARC: thymus and activation-regulated chemokine
